# Recurrent Pneumonia Due to an Appendiceal Mucinous Cystadenocarcinoma: A Rare Presentation of a Rare Malignancy

**DOI:** 10.4137/cmo.s1178

**Published:** 2009-03-02

**Authors:** Karin G. Gerritsen, Peter H. Slee, Thomas L. Bollen, Wim. van Hecke, Cornelis A. Seldenrijk, Ruth G. Keijsers, Vincent A. Duurkens

**Affiliations:** 1 Department of Internal Medicine, University Medical Centre Utrecht, University of Utrecht, Utrecht, The Netherlands.; 2 Department of Internal Medicine, St. Antonius Hospital, Nieuwegein, The Netherlands.; 3 Department of Radiology, St. Antonius Hospital, Nieuwegein, The Netherlands.; 4 Department of Pathology St. Antonius Hospital, Nieuwegein, The Netherlands.; 5 Department of Nuclear Medicine, St. Antonius Hospital, Nieuwegein, The Netherlands.; 6 Department of Pulmonology, St Antonius Hospital, Nieuwegein, the Netherlands.

**Keywords:** appendiceal neoplasms, mucinous adenocarcinoma, transdiaphragmatic lung invasion, recurrent pneumonia

## Abstract

Mucinous cystadenocarcinoma of the appendix is a rare malignancy. This is a report of a 74-year-old man who presented with recurrent pneumonia which turned out to be a postobstructive pneumonia complicating a large mucinous cystadenocarcinoma of the appendix with massive retroperitoneal and intrathoracic extension. Mucinous cystadenocarcinoma of the appendix is a low-grade malignancy characterized by expansive growth due to progressive accumulation of mucinous fluid produced by the cancer cells. The tendency of this tumor to expand massively is well demonstrated by this case. The unusual retroperitoneal location of appendix in this patient probably allowed the tumor to expand massively in the retroperitoneal space and the thoracic cavity. In addition to computed tomography, [^18^F]fluorodeoxyglucose positron emission tomography (^18^F-FDG PET) was used as an ancillary method for staging in this patient. The value of ^18^F-FDG PET in the diagnosis of mucinous cystadenocarcinoma of the appendix has not been determined yet, but it might be promising. The most common presentation of this tumor is abdominal pain or a palpable ileocoecal mass. To the knowledge of the authors, this is the first report of an appendiceal mucinous cystadenocarcinoma with expansion into the thoracic cavity presenting with recurrent pneumonia.

## Introduction

Primary malignancies of the appendix are rare with an age-adjusted incidence of 0.12 cases per 1,000,000 people per year. The five most common histologic types are mucinous adenocarcinoma, ‘colonic type’ adenocarcinoma, ‘malignant carcinoid’, goblet cell carcinoid and signet ring cell carcinoma.^1^ Mucinous adenocarcinoma is a low-grade well-demarcated malignancy characterized by expansive growth due to progressive accumulation of mucinous fluid produced by the cancer cells. This report describes a unique case of appendiceal mucinous cystadenocarcinoma with massive retroperitoneal expansion and direct transdiaphragmatic lung invasion presenting with recurrent pneumonia.

## Case Report

**Figure 1.**
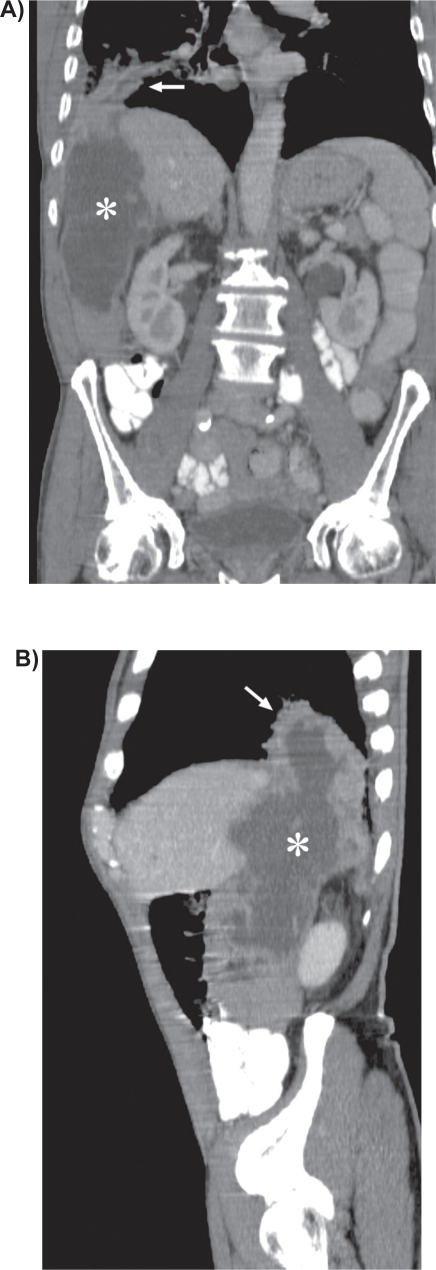
Coronal **A**) and sagittal **B**) reformatted CT images reveal the large cystic mass (asterix) extending from the region of the appendix retroperitoneally behind the liver into the right lower lobe (arrow).

**Figure 2.**
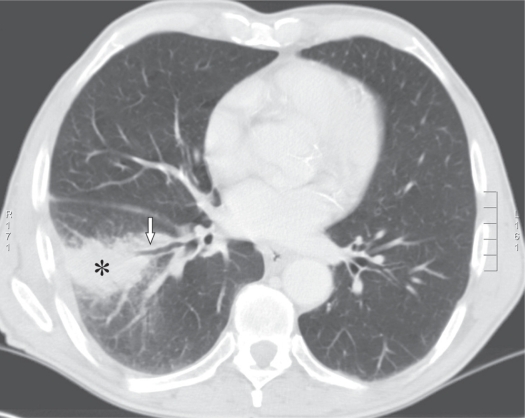
Axial CT image showing the consolidation in the right lower lobe (asterix) and the lateral basal segment of the right lower lobe bronchus (arrow).

**Figure 3.**
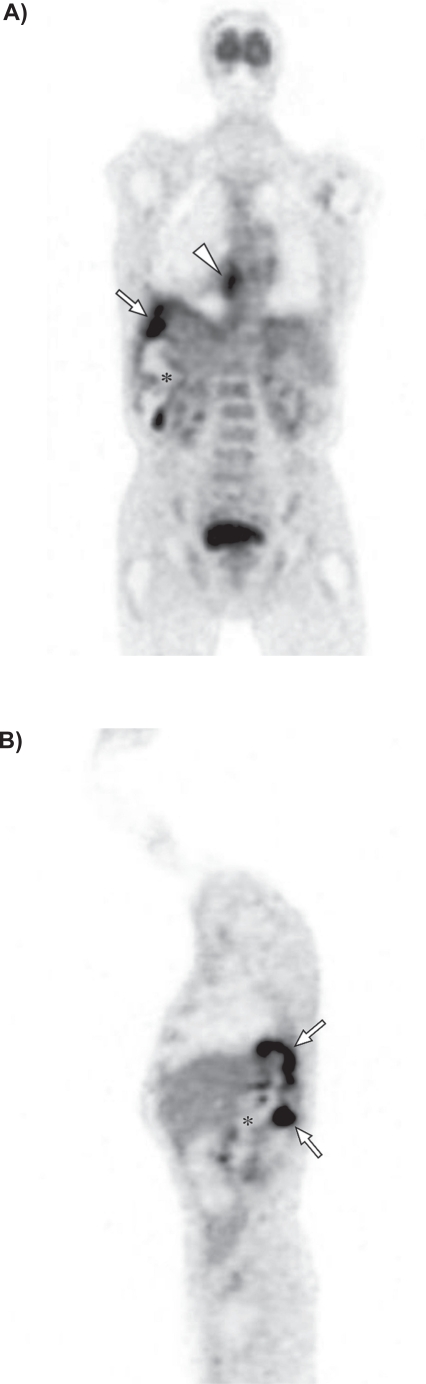
Coronal **A**) and sagittal **B**) ^18^F-FDG PET images show increased metabolic activity posteriorly in the right lower lobe of the lung extending through the diaphragm and liver into the right upper abdomen (arrows). The centre of the abdominal mass is photopenic, indicating the presence of fluid or necrosis (asterix). In addition, increased uptake is seen in the right hilum and mediastinum (arrowhead).

A 74-year-old, previously healthy man with an 8-pack-year history of cigarette smoking presented with a 9-month history of recurrent pneumonia and chronic right-sided thoracic pain. He was treated repeatedly with antibiotics. Laboratory investigation yielded a C-reactive protein of 139 mg/l (0 to 10 mg/l) and an erythrocyte sedimentation rate of 109 mm/h (0 to 7 mm/h). Chest radiograph showed consolidation in the lower lobe of the right lung. Due to the clinical history of recurrent pneumonia and the findings of the chest radiograph, postobstructive consolidation was considered. Bronchoscopy showed a stenosis at the entrance of the lateral basal subsegment of the lower lobe and purulent secretion. Cultures yielded *Morganella morganii* and *Proteus mirabilis*. Transbronchial biopsies and cytologic findings of bronchoalveolar lavage were nondiagnostic. Computed tomography (CT) of the chest and abdomen revealed a large cystic mass extending from the region of the appendix retroperitoneally behind the liver through the right hemidiaphragm into the lower lobe of the right lung with postobstructive consolidation (Figs. 1a, 1b and 2). To rule out colonic involvement of the mass, colonoscopy was performed which showed no abnormalities. [^18^F]Fluorodeoxyglucose positron emission tomography (^18^F-FDG PET) showed increased metabolic activity in the right abdomen extending through the liver into the right lung (Figs. 3a and 3b) and increased activity in the right hilum and mediastinum (Fig. 3a). The centre of the abdominal mass was photopenic, indicating the presence of fluid or necrosis. Ultrasound guided biopsy of this mass showed mucinous adenocarcinoma probably of bowel origin (Figs. 4a and 4b). Tumor cells contained mucin (Periodic Acid Schiff (PAS) diastase staining). Immunohistochemical staining of the atypical cells was positive for caudal type homeobox transcription factor 2 (CDX2) and cytokeratin 20 and negative for cytokeratin 7, a profile consistent with gastrointestinal tract origin. Cytological examination of fine-needle aspirate of mediastinal lymph nodes was not conclusive. Based on imaging and pathologic findings, the final diagnosis of mucinous cystadenocarcinoma of the appendix was made. Total resection of the tumor was considered impossible because the tumor was too large and expanded into the liver and thoracic cavity. On laparoscopy, no signs of pseudomyxoma peritonei were present and a palliative ileotransversostomy was performed. Systemic chemotherapy with oxaliplatin and capecitabine was offered, but the patient declined chemotherapy. One year after diagnosis the patient is doing reasonably well, except for chronic cough and recurrent pneumonia.

## Discussion

Mucinous cystadenocarcinoma of the appendix is an uncommon, low-grade malignancy with an incidence of approximately 0.044 cases per 1,000,000 people per year.^1^ Mucus-producing cells can cause massive growth due to large amounts of mucin and can spread intraperitoneally causing pseudomyxoma peritonei.^2^ The tendency to massive expansion is well demonstrated by this case. Clinical symptomatology of mucinous appendiceal adenocarcinomas is not specific. The most common presentation is a palpable ileocoecal mass or abdominal pain, which may simulate acute appendicitis. The course of the disease is often asymptomatic, even with large tumors.^3^–^5^ This patient had a unique initial presentation with recurrent pneumonia. It is well known that neoplasms may present as a nonresolving infiltrate. In a study of 35 adults with nonresolving pneumonia, 4 (11%) had a neoplastic disorder.^6^ However, direct transdiaphragmatic lung invasion of an infradiaphragmatic neoplasm is very rare. It has been described in primary retroperitoneal carcinoma.^7^ This patient had an unusual retroperitoneal location of the appendix, which has an incidence of 2.5%–7%.^8^ This retroperitoneal location probably allowed the tumor to expand massively in the retroperitoneal space and the thoracic cavity instead of the usual intra-abdominal expansion.

Patients with appendiceal mucinous cystadenocarcinoma show a great variation in survival. Five-year survival rates for all stages range from 45% to 75% in different series.^1^,^9^–^11^ The appropriate treatment is resection of the whole tumor and right hemicolectomy. There is a survival advantage for right hemicolectomy compared to appendectomy alone.^10^ If there is evidence of peritoneal dissemination, an aggressive therapeutic strategy is recommended, combining cytoreductive surgery with perioperative intraperitoneal chemotherapy. 3,12,13 In this patient, the growth into the liver and thoracic cavity made total resection impossible. The value of systemic chemotherapy and radiotherapy in mucinous adenocarcinoma is not known, because no randomized trials have been conducted.

In addition to CT, ^18^F-FDG PET was used as an ancillary method for staging in this patient. ^18^F-FDG PET is used in clinical oncology for a variety of tumors. However, the role in mucinous neoplasms is uncertain. One retrospective study found a sensitivity of only 59% for the detection of mucinous neoplasms originating from several organs in a group of 22 patients.^14^ This was explained by low tumor cellularity and overall abundance of mucin. In contrast, a prospective study to determine the value of ^18^F-FDG PET in the management of cystic tumors of the pancreas showed a 100% sensitivity and specificity in a subgroup of 21 patients with mucinous neoplasms.^15^ The value of ^18^F-FDG PET in mucinous cystadenocarcinoma of the appendix has not been determined yet, but it might be promising.

In summary, a unique case of recurrent pneumonia is presented here, caused by an appendiceal mucinous cystadenocarcinoma that expanded retroperitoneally and invaded the thoracic cavity.

## Figures and Tables

**Figure 4a:**
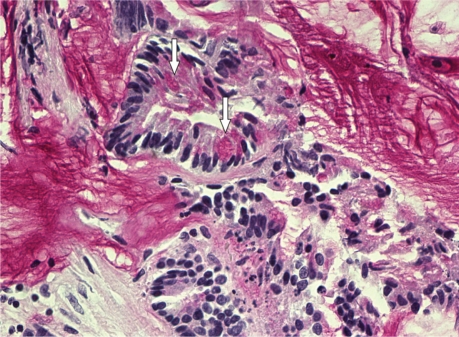
PAS diastase staining of the tumor showing atypical glands floating in a mucin background. Intracytoplasmic mucin globules are seen (arrows) (magnification 200×).

**Figure 4b:**
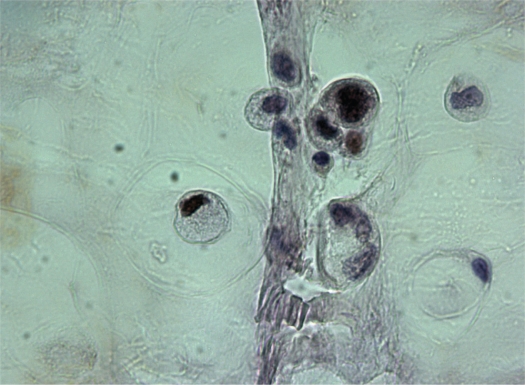
Immunohistochemical staining showing atypical tumor cells with nuclear expression of CDX2 (magnification 650×).
